# Delignification Effects on Indonesian Momala (*Homalium foetidum*) and Korean Red Toon (*Toona sinensis*) Hardwood Pore Structure and Sound Absorption Capabilities

**DOI:** 10.3390/ma14185215

**Published:** 2021-09-10

**Authors:** Eun-Suk Jang, Chun-Won Kang

**Affiliations:** Department of Housing Environmental Design and Research Institute of Human Ecology, College of Human Ecology, Jeonbuk National University, Jeonju 54896, Korea; esjang@sambosc.com

**Keywords:** delignification, NRC, momala, red toon, sound absorption capability

## Abstract

Among the various methods used to improve the sound absorption capability of wood, we focused on delignification in Indonesian momala (*Homalium foetidum*) and Korean red toon (*Toona sinensis*). We performed gas permeability, pore size, and porosity analyses and evaluated how the change in the pore structure affects the sound absorption capabilities. Results show that delignification increased the through-pore porosity and improved sound absorption capability in both species. In addition, the air gap in the rear space maximized the sound absorption of momala and the red toon. The noise reduction coefficient (NRC) of delignified momala (90 min) with a 3 cm air gap was 0.359 ± 0.023. This is approximately 154.6% higher than that of untreated momala without an air gap. The NRC of delignificated red toon (90 min) with a 3 cm air gap was 0.324 ± 0.040, an increase of 604.3% over untreated red toon without an air gap.

## 1. Introduction

There is much interest in using eco-friendly sound-absorbing materials to reduce noise pollution [1]. Most current eco-friendly sound-absorbing materials are natural fibers or natural fiber composites, using agricultural by-products [2,3,4,5]. Wood is a naturally porous material and can serve as an excellent eco-friendly and sustainable sound-absorbing material [6,7,8,9,10,11].

The porous sound-absorbing material uses the principle that sound energy is converted into heat energy, as sound waves vibrate as they strike the pores. Since it is easy to dissipate energy in the pores with many continuous channels, the sound absorption performance is superior [12,13]. Additionally, the lower density and higher porosity allow sound to enter into the material more easily, which can contribute to greater sound absorption [12].

The sound absorption capability of transverse sectional wood is correlated with permeability. In other words, woods with greater permeability tend to have an excellent sound absorption capability [14,15]. Wood permeability is determined by the vessel ratio and macroporous structure [16]. Macroporous structures in solid materials are classified into through-pore, blind-pore, and closed-pore categories, according to the physical shape [17]. Jang et al. [18] suggested using gas pycnometry and capillary flow porometry to classify wood porosity into through-pore porosity, blind-pore porosity, and closed-pore porosity categories, with a higher permeability correlating to higher through-pore porosity.

Tyloses—blind or closed pores—inside wood vessels most hinder its longitudinal permeability [14,19,20,21,22]. In order to eliminate these obstacles, various methods, such as heat treatment, microwave treatment, ultrasonic treatment, steam explosion treatment, and delignification treatment, have been studied [8,23,24,25,26].

Our study examines delignification treatment. Wood delignification can significantly increase porosity because it creates micro pores in the fibers [27]. Kang et al. [8] reported that, after *Larix kaempferi* delignification, intercellular substances were ejected, and various small cracks on the surface were observed. Through this anatomical change, gas permeability and sound absorption capability improved. They confirmed that the pore structure of wood was changed through delignification treatment, which improved the sound absorption capability. There are few studies, however, that show how delignification affects through-pore, blind-pore, and closed-pore shapes.

Our study examines how the pore shape of wood is changed after delignification and how these changes affect the gas permeability and sound absorption capability. According to previous research, an air gap applied to the rear side of the porous sound absorber improves the low-frequency sound absorption capability [13]. In this study, we investigate changes in the sound absorption capabilities, using an air gap at the back of a delignified sample.

## 2. Materials and Methods

### 2.1. Specimen Preparation

**Figure 1:**
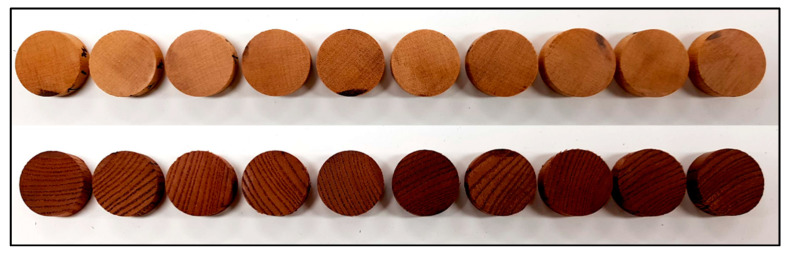
Sample preparation of momala (**up**) and red toon (**down**).

We used Indonesian momala (*Homalium foetidum*) and Korean red toon (*Toona sinensis*) wood for this study. Each specimen was supplied with 10 cm × 10 cm × 100 cm air-dried lumber from the Korean wood market (Saehan timber, Ilsan, Korea). From each species of lumber, we manufactured cylindrical samples with a 2.9 cm diameter and a 1 cm thickness and measured a 7% moisture content (MC) by oven dry testing. We verified that samples were without cracks or knots. We selected 10 cylindrical samples from each species for measuring the gas permeability, pore size, porosity, and sound absorption coefficient (Figure 1); then, we prepared one sample from each species for analysis with SEM (Scanning Electron Microscope). The average density of 10 samples was 0.80 ± 0.02 g/cm^3^ for momala and 0.57 ± 0.02 g/cm^3^ for red toon. It was difficult to find growth rings in the momala specimens. Red toon had about 10–15 growth rings.

### 2.2. Delignification Treatment

For the delignification treatment, we placed the samples in a boiling aqueous solution of 2.5 M NaOH and 0.4 M Na_2_SO_3_ mixture [28], and decompressed them at −0.1 Mpa, 25 °C for 30 min in a vacuum chamber. Afterward, we washed the samples in ultrapure water for 30 min, air-dried them for 2 weeks, and measured their sound absorption coefficients. We performed another round of 30 min delignification treatment and measured the sound absorption coefficients after washing and drying. Delignification was performed for up to 90 min. For untreated samples, we measured the sound absorption coefficient every 30 min. For both the treated and untreated samples, we performed SEM and gas permeability, pore size, and porosity analyses after 90 min.

### 2.3. SEM Image Analysis

As in the previous studies, wood specimens require pretreatment for SEM observation [18,23,29]. First, we immersed the samples in water to soften them, dried them, and pre-treated them with a gold ion coating. We used SEM at 200× magnification (model: Genesis-1000, Emcrafts, Sungnam, Korea) to observe the transverse and radial sections before and after the delignification treatment.

### 2.4. Measurement of Gas Permeability and Pore Size

We used a capillary flow porometer (model: CFP-1200AEL, Porous Materials Inc., Ithaca, NY, USA) to measure the gas permeability and pore size. We increased the air pressure by 0–1 bar for gas permeability analysis and measured the pore size, using a ‘dry up/wet up’ method [30], according to ASTM F316 [31]. The results were calculated automatically, using capillary flow porometer software (Capwin ver. 6.74.110).

### 2.5. Porosity Classification by Pore Shape

**Figure 2:**
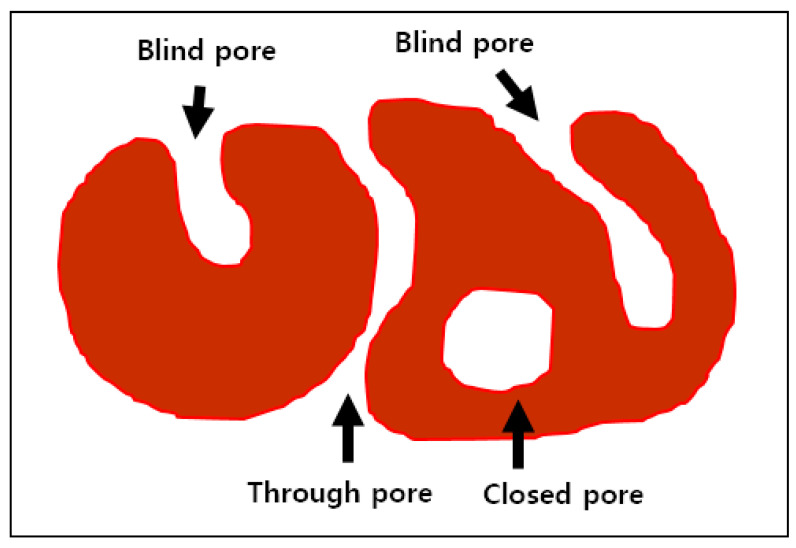
Pore classification by physical shape (Total porosity = Open-pore porosity + Closed-pore porosity; Open-pore porosity = Through-pore porosity + Blind-pore-porosity).

Figure 2 provides pore classification by physical shape. “Blind pore” is a shape in which one side is blocked. “Closed pore” is formed like a bubble inside the material, limiting the connection to the outside. On the other hand, “through pores” are open on both sides so that the fluid can penetrate the inside of the material. We used the same pore shape porosity determination methods as in previous studies [18,23]. We calculated the porosity, *ϕ* from the bulk density, *ρ*_b_, and true density, *ρ_t_* (Equation (1)), of the wood. We assumed the wood substance density to be 1.5 g/cm^3^ [32,33].

(1) $$\phi =\left(1-\frac{\rho_{b}}{\rho_{t}}\right)\times 100\;$$

where *ρ_t_* is assumed to be 1.5 g/cm^3^ [32,33].

We obtained the open-pore porosity, *ϕ*_open,_ of the cylindrical sample, using a gas pycnometer (model: PYC-100A-1, Porous Materials Inc., Ithaca, NY, USA). The closed-pore porosity, *ϕ*_closed_ was obtained from the difference between the open-pore porosity and total porosity (Equation (2)):

(2) $$\phi_{\mathrm{closed}}=\;\phi_{\mathrm{total}}-\phi_{\mathrm{open}}$$

To obtain blind-pore porosity, *ϕ*_blind_, and through-pore porosity, *ϕ*_through_, from open-pore porosity, *ϕ*_open_, we immersed cylindrical samples in Galwick solution (Porous Materials Inc., Ithaca, NY, USA) and vacuumed for 15 min. We assumed that the Galwick solution only entered open pores. We placed a sample in the capillary flow porometer chamber and applied 0–6 bar air pressure. The Galwick solution only extruded through the through pores because they are open on both ends, while the blind pores are open only on one end. We recorded the sample weight before and after wetting with the Galwick solution and applying air pressure. The difference yields the weight of the Galwick solution remaining in the blind pores. Equation (3) is used to calculate the blind-pore porosity:

(3) $$\phi_{\mathrm{blind}}\;\left(\%\right)=\left(\frac{\left(m_{1}-m_{0}\right)/\rho_{G}}{V_{sample}}\right)\times 100$$

where *m*_0_ = sample weight before wetting with the Galwick solution, *m*_1_ = sample weight before wetting with the Galwick solution, *V_sample_* = cylindrical sample volume and *ρ_G_* = Galwick density (1790 kg/m^3^).

As shown in Equation (4), the through-pore porosity is the difference between the open-pore porosity and blind-pore porosity:

(4) $$\phi_{\mathrm{through}}\;\left(\%\right)=\phi_{\mathrm{open}}-\phi_{\mathrm{blind}}$$

### 2.6. Measurement of Sound Absorption Coefficient

There are two major methods for evaluating the capability of sound absorbing materials: the reverberation room method (ISO 354) [34] and the impedance tube method (ISO 11534-2) [35]. In this study, we used a small impedance tube (model: Type 4206, B&K, Nærum, Denmark) that can measure the absorption coefficient quickly even with a small sample (diameter 29 mm). We measured the sound absorption coefficient at intervals of 8 Hz from 0 to 6400 Hz. In addition, we introduced a noise reduction coefficient (NRC) to evaluate the sound absorption capability as a single index. We calculated NRC as the average of the absorption coefficients at 250 Hz, 500 Hz, 1000 Hz, and 2000 Hz.

### 2.7. Multiple Regression Analysis

We performed multiple regression analysis to investigate the effects of delignification treatment time and the air gap on NRC. Equation (5) shows our multiple regression model.

*Y* = *α*_0_ + *β*_1_*X*_1_ + *β*_2_*X*_2_ + *β*_3_*D*_1_ + *ε* (5)

where *Y* is the NRC, *X*_1_ is the treatment time, *X*_2_ is the air gap distance, *D*_1_ is the wood dummy variable (1 for red toon, otherwise 0), *α*_0_ is the constant (intercept term), and ε is the residuals (error term).

We set the NRC as the dependent variable, and treatment time and the air gap were set as independent variables. We also set up a dummy variable to investigate the difference in sound absorption according to species, and evaluated the multi co-linearity between independent variables by analyzing variance inflation factors (VIF) for all of the independent variables. If the estimated VIF < 10, the multi co-linearity is not serious [18]. Multiple regression analysis was performed using statistics software (model: IBM SPSS statistics v25, Armonk, NY, USA).

## 3. Results and Discussion

### 3.1. Morphology Analysis

**Figure 3:**
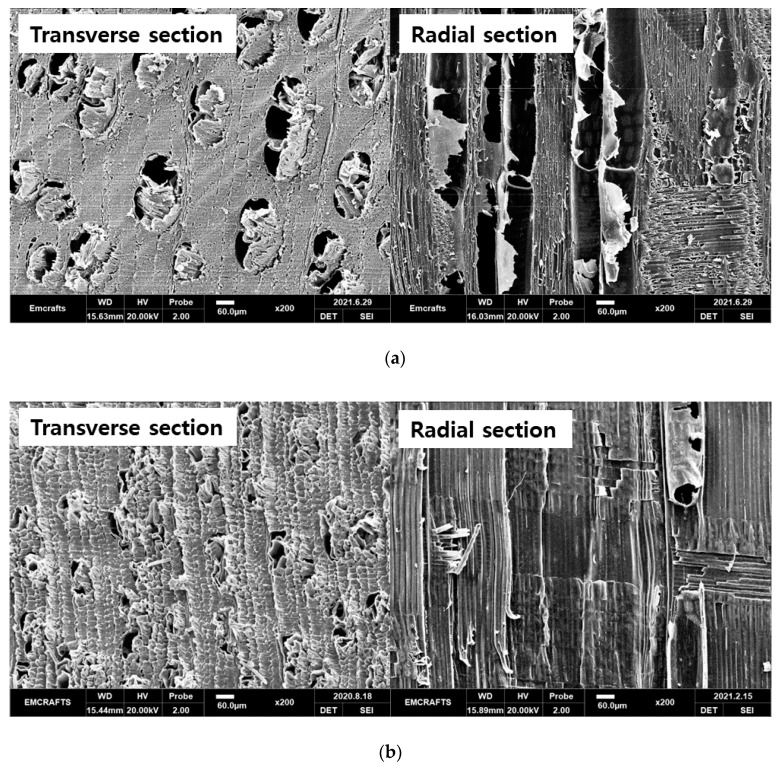

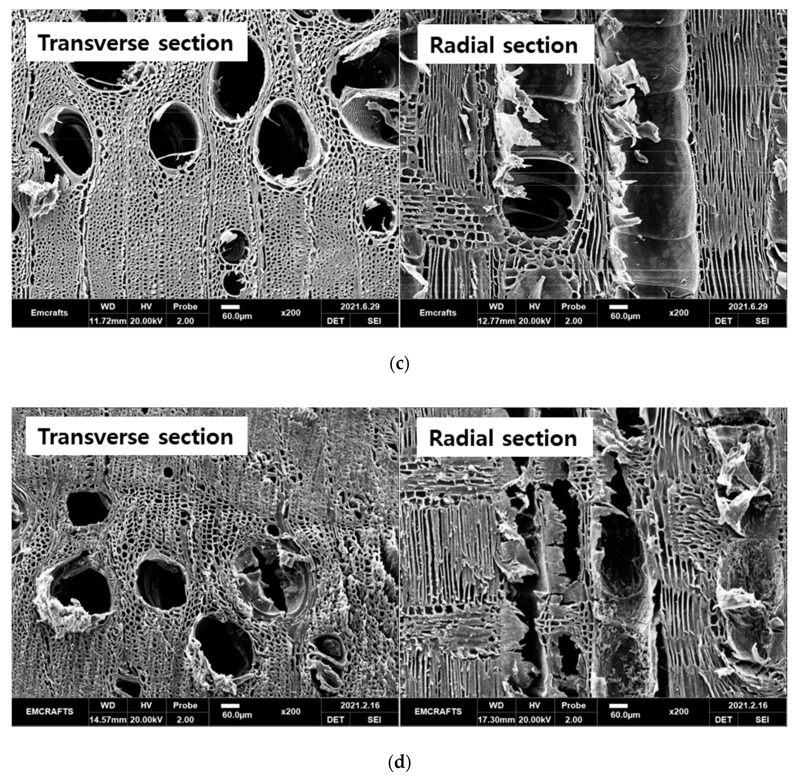
SEM images of momala and red toon before and after delignification treatment: (**a**) Untreated momala; (**b**) Delignificated momala; (**c**) Untreated red toon; (**d**) Delignificated red toon.

Figure 3 provides SEM images of momala and red toon wood before and after the delignification treatment. Momala showed a diffuse-porous wood shape with evenly distributed vessels, while the red toon showed a ring-porous wood shape with large vessels distributed in the spring wood.

In the delignification-treated samples, the sample surfaces were crushed easily when cut with a microtome, and the overall cell walls appeared to be thinned. Accordingly, the vessel elements did not maintain their shape. A number of surface checks was observed in the delignification-treated red toon. As the specimen contracted overall, the vessel size seemed rather small.

### 3.2. Changes of Gas Permeability, Pore Size, and Porosity after Delignification

**Figure 4:**
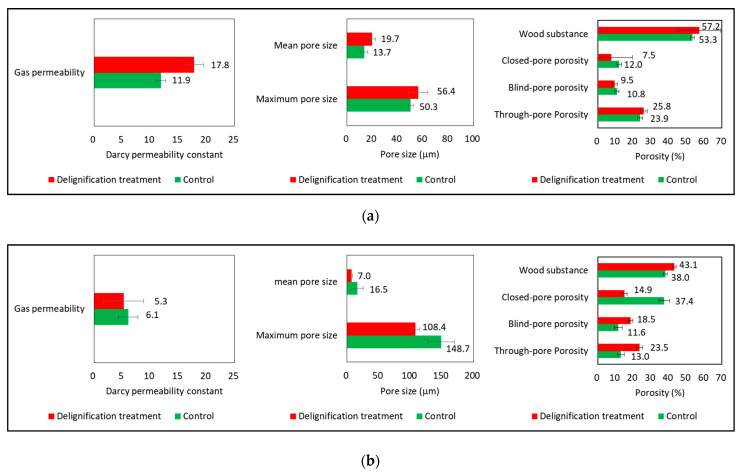
Gas permeability, pore size, and porosity analysis before and after delignification treatment of momala and red toon: (**a**) Momala; (**b**) Red toon.

Figure 4 shows the momala and red toon gas permeability, pore size, and porosity analysis before and after delignification treatment. The gas permeability of delignified momala increased by 50.0%, compared to untreated momala wood (t = −17.435, *p* < 0.001). After delignification, the maximum pore size increased by 5.7% (t = −2.447, *p* < 0.05), the mean pore size increased by 43.1% (t = −4.915, *p* < 0.05), and the through-pore porosity increased by 8.4% (t = −3.331, *p* < 0.01).

The increases in pore size and through-pore porosity are due to the increase in void volume from lignin removal; the gap between the cell walls widened as the wall became thinner. Gas permeability increased due to the change in the pore structure of momala.

The difference in red toon gas permeability between delignified and untreated samples was not statistically significant (t = 1.455, *p* = 0.179). After delignification, the maximum pore size decreased by 27.1% (t = 5.262, *p* < 0.001), and the mean pore size decreased by 57.3% (t = 3.265, *p* < 0.001).

As lignin was removed, we expected an increase in pore size, but results showed the opposite effect. Immediately after delignification of red toon, the pore size increased. However, we think the pore size ultimately decreased from contraction during the re-drying process.

**Figure 5:**
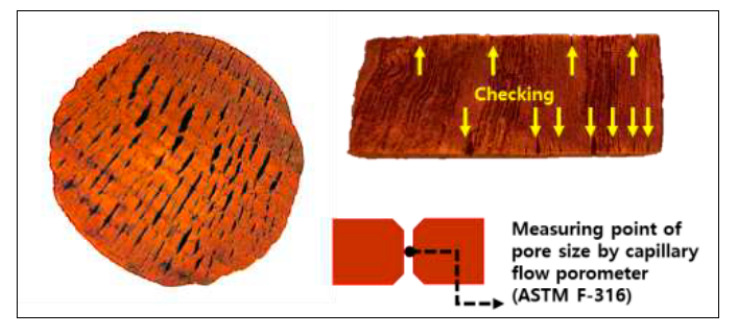
Checking of delignification-treated red toon and measuring point of pore size by capillary flow porometer.

After delignification, the through-pore porosity increased by 81.2% (t = −10.475, *p* < 0.001). Although the overall red toon pore size decreased, the through-pore porosity increased from checking. As shown in Figure 5, checking occurs on the surface but not in the interior. Surface checking is connected vertically through constricted vessels and can be considered a through pore. The pore size was assessed, using capillary flow porometry in the contracted portion of the through pore only [18,23], resulting in the smaller pore size measurements. The reason that the gas permeability remained unchanged after delignification is because the through-pore porosity increase was offset by the pore size decrease. The through-pore porosity and pore size are major parameters that determine gas permeability [18,23].

### 3.3. Changes of Sound Absorption Coefficient after Delignification

**Figure 6:**
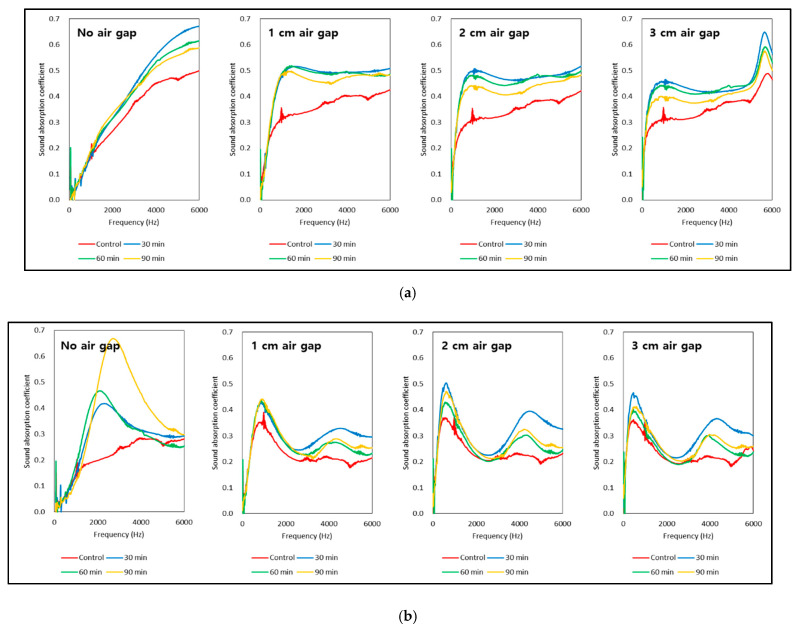
Sound absorption coefficient curve before and after delignification treatment of momala (**a**) and red toon (**b**) wood with an air gap: (**a**) Momala; (**b**) Red toon.

Figure 6 shows the sound absorption coefficient curves of momala and red toon before and after delignification with an air gap. Overall, the sound absorption property of the untreated species showed a tendency for low sound absorption in the low-frequency band with an increase in sound absorption coefficient with movement to the high-frequency band. This is a typical sound absorption characteristic of porous materials [3,13].

After delignification of the momala wood, the sound absorption coefficient at high frequencies above 2000 Hz gradually increased. In red toon wood, however, a peak value appeared between 2000 and 4000 Hz. The two species showed different absorption patterns in the presence of an air gap. The sound absorption coefficient of momala increased in the low-frequency band because of the diffuse pores that absorb sound waves via a large number of vessels. The red toon wood, however, showed a peak value in the low-frequency band because of its ring-porosity, with large vessels arranged in the springwood that allow the passage of and sound waves to absorb resonance in the rear space.

As the delignification treatment time increased, the sound absorption coefficient in momala at high frequencies was decreased because the shrinkage occurring during re-drying increased. The red toon peak sound absorption coefficient at low frequency increased as the delignification treatment time increased because of increased checking during re-drying.

Table 1 lists the NRC of momala and red toon before and after the delignification treatment with an air gap. The NRC of delignification-treated momala (90 min) was 0.160 ± 0.010, which is 13.5% higher than that of untreated momala wood (t = −5.770, *p* < 0.001, paired *t*-test). With a 3 cm air gap, the delignification-treated momala (90 min) NRC was 0.359 ± 0.023, a 154.6% (t = −24.422, *p* < 0.001) increase over untreated momala with no air gap.

For momala wood, the overall sound absorption capability is significantly improved by applying an air gap in addition to the delignification treatment.

The NRC of delignification-treated red toon (90 min) was 0.169 ± 0.016, which is 267.4% higher than that of untreated red toon (t = −26.409, *p* < 0.001). With a 3 cm air cavity, the NRC of delignification-treated red toon was 0.324 ± 0.040, which is a 604.3% (*t* = −18.087, *p* < 0.001) increase over that of untreated red toon with no air gap.

Table 2 shows the results from the multi-regression analysis. The F-value of the multiple regression model was 262.505 with *p* < 0.001, demonstrating statistical significance. The VIF value indicates no problem with multicollinearity in the model.

Delignification treatment time (*β*_1_) and an air gap (*β*_2_) are significant in the positive (+) direction at a 1% significance level. The adjusted R^2^ was 71.1% for the regression model. This means that an increase in the delignification time and addition of an air gap improved the NRC, with a greater NRC increase for red toon than in momala.

## 4. Conclusions

We applied a delignification treatment to momala and red toon wood to create a more permeable pore structure. This change led to improvement of the sound absorption capability. When we introduced an air gap into the rear space, it maximized the sound absorption capability for both momala and red toon woods. The NRC for delignified momala wood for 90 min without air gap was 0.160 ± 0.010, 0.338 ± 0.028 for a 1 cm air gap, 0.369 ± 0.020 for a 2 cm air gap, and for 3 cm air gap it was 0.359 ± 0.023, a 154.6% increase over that of untreated momala with no air gap. The NRC for delignified red toon wood for 90 min without an air gap was 0.169 ± 0.016, 0.280 ± 0.028 for a 1 cm air gap, 0.329 ± 0.044 for a 2 cm air gab, and for a 3 cm air gap, it was 0.324 ± 0.040, a 604.3% increase over that of untreated red toon wood with no air gap. In the future, pore structure improvements could be made by applying delignification treatments to other species. Delignified wood is an eco-friendly and sustainable porous, sound-absorbing material.

## Figures and Tables

**Table 1:** NRC values of momala and red toon before and after delignification treatment and addition of an air gap.

Air Gap (cm)	Delignification Time							
	Control		30 min		60 min		90 min	
	Momala	Red Toon	Momala	Red Toon	Momala	Red Toon	Momala	Red Toon
Non	0.141	0.046	0.152	0.163	0.158	0.188	0.160	0.169
	(0.004)	(0.011)	(0.009)	(0.009)	(0.014)	(0.013)	(0.010)	(0.016)
1	0.262	0.246	0.346	0.278	0.344	0.281	0.338	0.280
	(0.018)	(0.011)	(0.022)	(0.030)	(0.018)	(0.027)	(0.028)	(0.028)
2	0.275	0.289	0.405	0.351	0.398	0.317	0.369	0.329
	(0.016)	(0.027)	(0.023)	(0.021)	(0.024)	(0.046)	(0.020)	(0.044)
3	0.292	0.293	0.406	0.347	0.394	0.316	0.359	0.324
	(0.022)	(0.031)	(0.030)	(0.046)	(0.023)	(0.047)	(0.023)	(0.040)

Note: The parentheses are the standard deviation.

**Table 2:** Results of multi-regression analysis.

*Y* = *α*_0_ + *β*_1_*X*_1_ + *β*_2_*X*_2_ + *β*_3_*D*_1_ + *ε*				
Variables	Coeff.	Std. Coeff.	t-stat.	VIF
*α*_0_	0.149	-	25.665 **	
*X*_1_	0.001	0.140	4.666 **	1.000
*X*_2_	0.060	0.825	27.406 **	1.000
*D*_1_	0.019	0.115	3.826 **	1.000
Adj. R^2^	0.711			
F-Value	262.505			
*p*-value	<0.001			

Notes: ** represent significance at the 1% levels Denotations of variables are as follows; *Y*: NRC, *X*_1_: Delignification treatment time, *X*_2_: Air gap distance, *D*_1_: Wood dummy variable (1: if the wood is red toon, otherwise 0), *α*_0_: Constant (Intercept term); and *ε*: Residuals (Error Term).

## Data Availability

Not applicable.
